# White-opaque flowable composite liner as a depth marker in composite restorations prevents tooth substance loss in filling removal: a randomized double-blinded in vitro study

**DOI:** 10.1007/s00784-021-04244-5

**Published:** 2021-10-29

**Authors:** Thomas Gerhard Wolf, Natalie Dekert, Guglielmo Campus, Claus-Peter Ernst

**Affiliations:** 1 Department of Restorative, Preventive and Pediatric Dentistry, School of Dental Medicine, University of Bern, Freiburgstrasse 7, CH-3010 Bern, Switzerland; 2 Department of Periodontology and Operative Dentistry, University Medical Center, Johannes Gutenberg-University Mainz, Mainz, Germany; 3 Department of Surgery, Microsurgery and Medicine Sciences, School of Dentistry, University of Sassari, Sassari, Italy; 4 Department of Pediatric, Prophylaxis Dentistry and Orthodontics, School of Dentistry, Sechenov First Moscow State Medical University, Moscow, Russia; 5 medi+ Zahnärztliche Praxisklinik, Mainz, Germany

**Keywords:** Composite, Depth marking, Restoration replacement, Tooth substance loss, Time requirement, White-opaque liner

## Abstract

**Objectives:**

Removal of esthetic restorations leads to loss of tooth structure and the extent of the loss is difficult to estimate due to exact-shade matching. This randomized double-blinded in vitro study aimed examining the influence of a white-opaque flowable composite depth marker as an optical removal aid for tooth substance preservation and shortened restoration removal time.

**Materials and methods:**

Class II cavities (*n* = 100) in extracted healthy mandibular molars (*n* = 50, two runs) were prepared, filled, and the restoration removed. Tooth weight and volume (before and after) and removal time were measured and remnants visually documented. An optimal tooth shade-matched flowable composite liner was used as control.

**Results:**

Tooth structure loss was significantly lower using a white-opaque liner. Mean values for volume/weight loss were 0.037 ± 0.030 g and 0.016 ± 0.005 cm^3^ (*p* < 0.01) for white-opaque liner; 0.067 ± 0.000 g and 0.028 ± 0.003 cm^3^ (*p* < 0.01) for tooth-colored composite. Removal time and number of pulp chamber perforations showed no significant differences (*p* = 0.80).

**Conclusions:**

Within the limitations of this randomized double-blinded in vitro study, the use of a white-opaque flowable liner as a depth marker may provide the practitioner a visual aid in the replacement of a composite restoration and may protect against tooth structure loss.

**Clinical relevance:**

When restoration replacement is indicated, removal of esthetic restorations often causes tooth structure loss due to difficult optical color matching. Using a white-opaque flowable liner as a depth marker clinically aids in restoration removal and protects against tooth structure loss.

## Introduction

Recently, an increased interest in tooth-colored restorations has been noticed and attributed to medical and esthetic reasons [1–4]. In the European Union (EU), this trend has been strengthened by the call for phase out of dental amalgam by 2030 [5]. The decision has been made despite the conclusion of the Scientific Committee on Emerging and Newly Identified Health Risks (SCENIHR) at the EU Commission, although only minor health risks are associated with amalgam as well as other dental restorations [6]. However, the increased use of composite resin is of concern when one considers the longevity of such restorations [7] compared to amalgam and the common practice of replacement instead of repair of restorations adopted by dental providers. Replacement of restorations has accounted for more than half of the restorations placed by dental providers despite the knowledge that repairs can increase survival of restorations and be favorable in many aspects [8, 9]. While continuous education of dental providers in regard to the repair of dental restorations is necessary, procedures that promote minimally invasive dentistry in regard to protection against overextension of preparations should be studied. It is often difficult to discern between tooth structure and tooth-colored materials due to imperceptible transitions between structures [10]. The use of a liner or base to aid in the differentiation between composite resin and dentin is a possibility to be explored. It is known, however, that the presence of a glass ionomer liner or base may have a negative effect on the survival of restorations [3, 4]. The replacement of restorations still accounts for more than half of the restorations used by dentists with a trend towards the increased use of resin composites in recent years [10]. With modern dentistry’s focus on minimally invasive work, there is a desire to guard against the unnecessary or iatrogenic expansion of preparation dimensions [11], which is extremely difficult today due to optimally color-matched plastic restorations; because of low contrast, confidently differentiating between restoration material and tooth structure is not easy even for dentists with many years of professional experience [12]. This in vitro study evaluated the ability of a white-opaque flowable composite resin liner used as a depth marker in class II cavities prior to composite resin placement as a means to preserve tooth structure in case of restoration replacement. The study was designed to test the null hypothesis that a white-opaque flowable composite resin liner would not increase the extension of the preparation with a consequently unnecessary removal of dentin compared to preparations completed using a conventional technique/tooth-colored flowable liner.

## Materials and methods

### Experimental design and power analysis

This study was performed with an in vitro double-blind case–control design. Sample size calculation to obtain interpretable results was performed using OpenEpi: Open Source Epidemiologic Statistics for Public Health, version (www.OpenEpi.com). Considering as significant a 15% difference between groups [13]—with values of 40% and 15% for the case group (white-opaque flowable composite liner) and the control group (non-white-opaque flowable composite liner = tooth-colored flowable composite liner), respectively, and a 95% probability of obtaining a significant difference between groups at the 5% level—the resulting number of cavity preparations per group was set at 47.5.

### Tooth selection

From a pool of extracted molars stored in a 3% chloramine solution from the so-called excess material from the dental school of the University Medical Center of the Johannes Gutenberg University in Mainz (Germany), 50 healthy caries- and restoration-free mandibular molars were randomly selected (contract general terms [AVB], §14 Organ transplantation/further use of body material) and could be used for medical research without any additional approval of the local ethics committee. Written informed consent was obtained from each adult person for the use of the excess material for research purposes. The selection criteria were intact tooth crown, no restoration, no evidence of fracture of coronal or radicular caries, and absence of endodontic treatment.

### Specimen preparation

Soft and hard tissues as well as calculus were removed with a universal curette (S413/414, Hu-Friedy Manufacturing, Co., LLC, Chicago, IL, USA). Teeth were polished with a goat hair brush (Polirapid—Dr. Montemerlo GmbH & Co. KG, Singen, Germany) and pumice stone of medium grain size (Ernst Hinrichs Dental GmbH, Goslar, Germany). Subsequently, they were stored in distilled H_2_O in order to obtain good shade matching. The teeth were embedded with a composite material that was placed in polyethylene tubes (Carl Roth GmbH + Co. KG, Karlsruhe, Germany) using the increment method to fix the dental crown from the enamel–cement interface and thus isolate the roots. Each layer of the base was polymerized for 20 s (according to manufacturer power density > 1400 mW/cm^2^, wavelength 385–510 nm, Translux 2Wave, Kulzer GmbH, Hanau, Germany). At the bottom of the reference bodies, numbers were engraved with a diamond (mds citoMant, Höhr-Grenzhausen, Germany); afterward, the samples were stored in distilled H_2_O.

One cavity was prepared for each tooth; the mesial–occlusal–distal (MOD) class II cavity of mandibular molars was simulated by preparing a standardized preparation design [14] and using a pear-shaped fine/coarse diamond bur (X 234-012f, mds citoMant, Höhr-Grenzhausen, Germany). The occlusal box of the preparation was 3 mm (± 0.4 mm) in buccal–lingual width with a depth of at least 2 mm (± 0.4 mm) from the deepest point of the fissure. The buccal–lingual distance of the proximal box was 4 mm (± 0.5 mm) and 2 mm (± 0.4 mm) to central on each tooth side (mesial/distal). The distance of the cavity to enamel–cement junction was at least 1 mm (± 0.4 mm). The cavities were measured and checked for the specified reference ranges using a digital caliper (HSL 246–15, Karl Hammacher GmbH, Solingen, Germany).

### Testing procedure

**Fig. 1:**
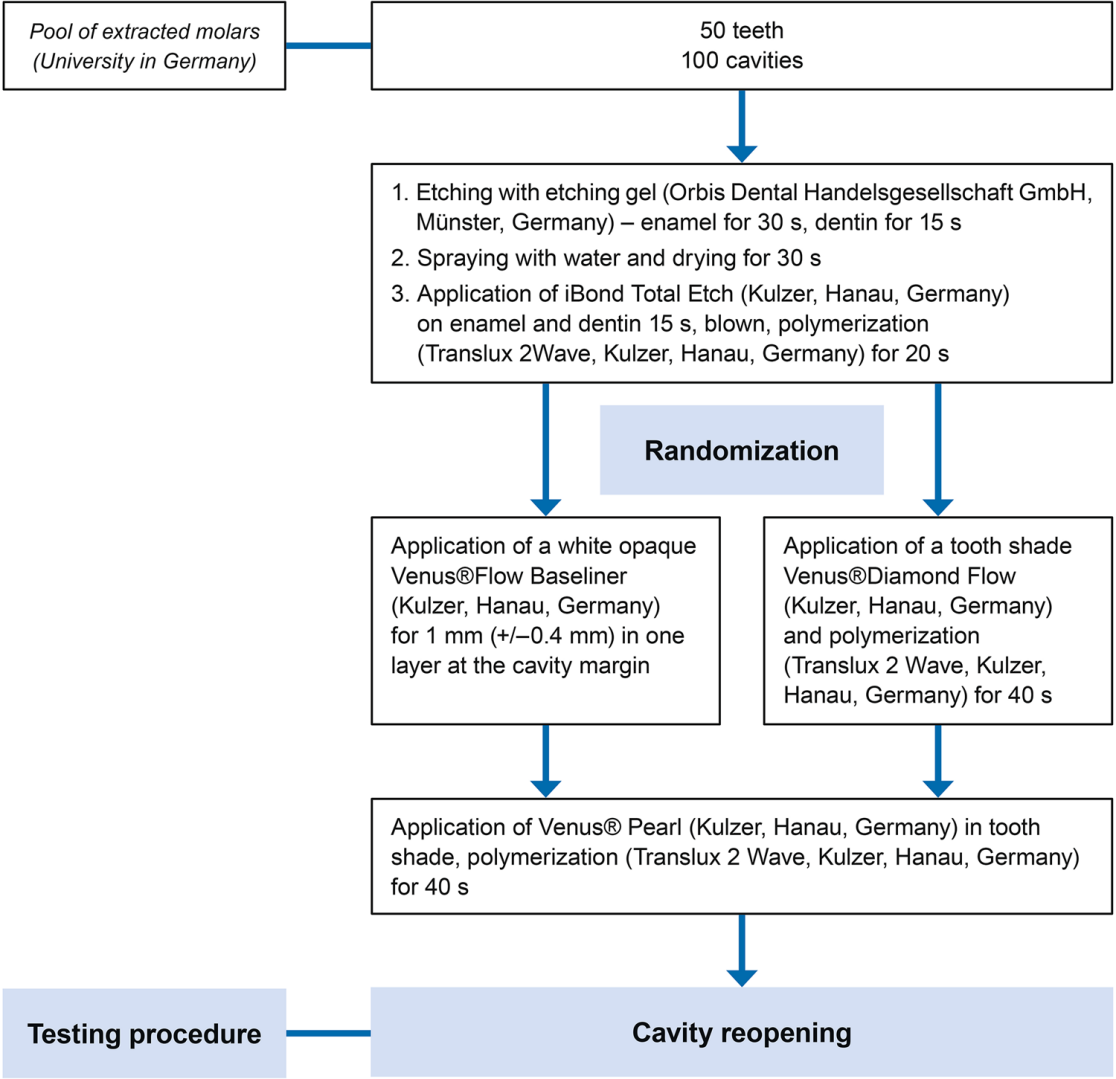
Flow chart with procedure for preparing the specimens and the evaluation

**Fig. 2:**
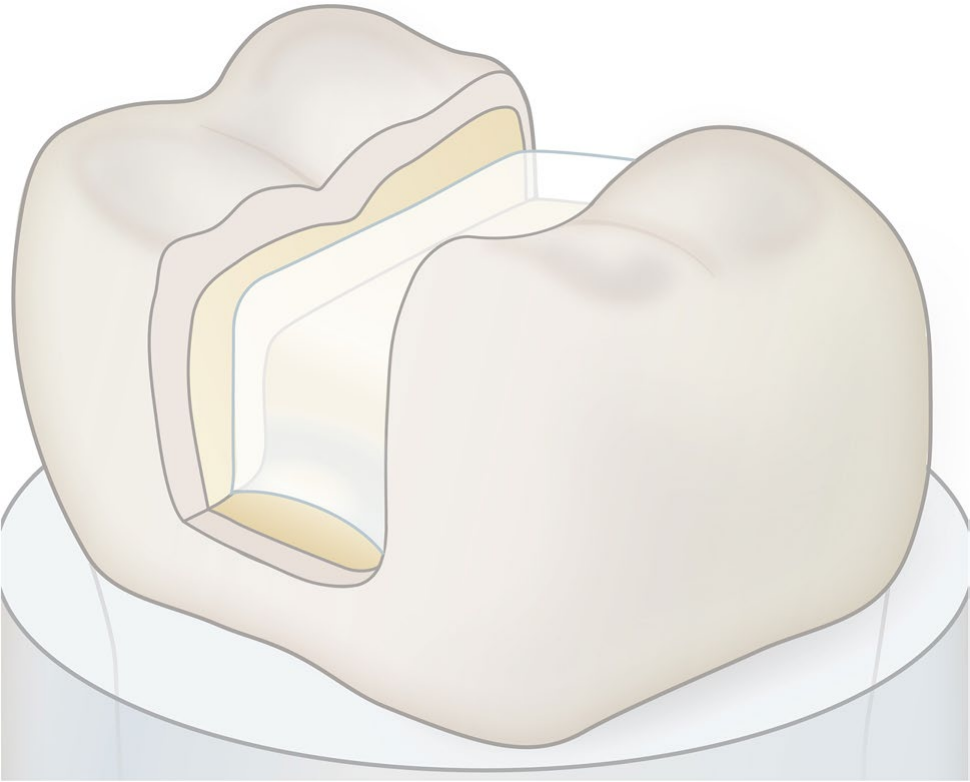
Liner application in the bottom of the prepared tooth cavity

**Fig. 3:**
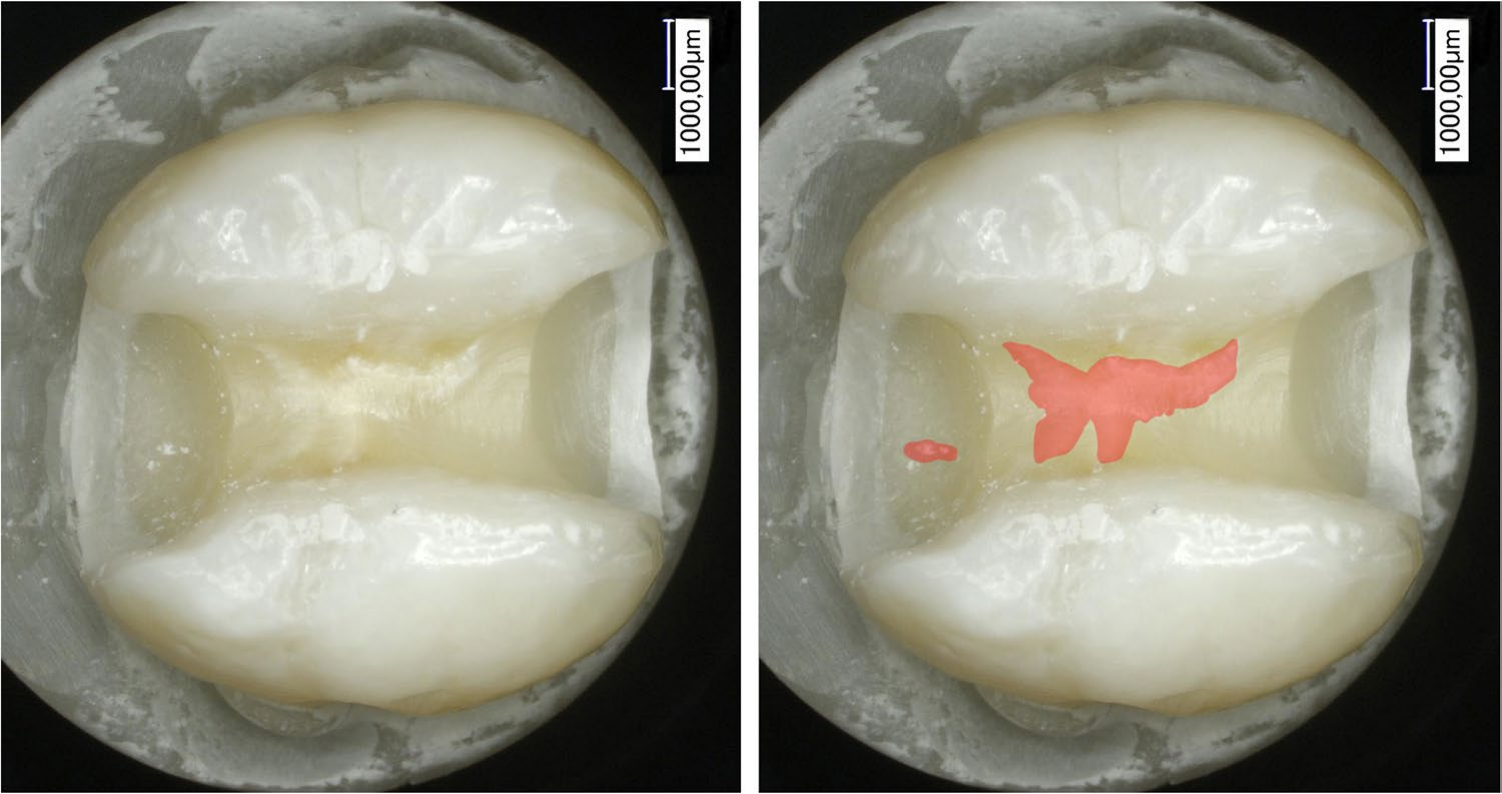
The remaining composite (tooth-colored liner)

All materials were used according to the manufacturer’s recommendations. The teeth were randomly divided into two groups of 25 teeth (total restorations = 25) each performing two experimental runs (each experimental run = 50 restorations) so that both groups (case and control) consisted of 50 teeth (total restorations = 100) (Fig. 1). Each procedure was repeated two times. Before the cavity preparation, the tooth-color was exactly determined in a light chamber (Atlas Variolux, Atlas Material Testing Technology, Mount Prospect, IL, USA), which was carried out under artificial and natural light with the color ring for Venus® Pearl (Kulzer GmbH, Hanau, Germany). The prepared cavities were digitally catalogued under standardized conditions (Canon EOS 5D Mark III, Canon, Öta, Tokyo, Japan) and then weighed to determine the tooth structure after restoration removal (Precisa 320 XT, Dietikon, Switzerland), and the volume was determined in a gas pycnometer (Accu Pyc H 1340 Micromeritics, Aachen, Germany). The cavities (Fig. 1) were etched (37% phosphoric acid, Orbis Dental Handelsgesellschaft GmbH, Münster, Germany)—enamel for 30 s and dentin for 15 s, rinsing with water and drying for 30 s. Then iBond Total Etch (Kulzer GmbH, Hanau, Germany) was used on enamel and dentin for 15 s, blown until no more movement of the material was noticeable and polymerized for 20 s (Translux 2Wave, Kulzer GmbH, Hanau, Germany). Then, after randomization, either a tooth shade Venus® Diamond Flow (Kulzer GmbH, Hanau, Germany) or a white-opaque Venus® Flow Baseliner (Kulzer GmbH, Hanau, Germany) was applied in one layer (1 mm) at the interface of tooth and restoration and polymerized for 40 s (Fig. 2). Venus® Pearl (Kulzer GmbH, Hanau, Germany) was then applied in the tooth shade (for the control group, the same color as the flowable tooth-colored liner was used) using the increment technique and layers of a maximum of 2 mm were applied and polymerized for 40 s each. Subsequently, the teeth were stored in a container in H_2_O and then handed to a single operator (N.D.) to remove the restorations. The time was measured with a stopwatch (Oregon Scientific, Gennevilliers, France) from the beginning to the end of the restoration removal. Restoration removal was first carried out with a fine/coarse diamond bur (X 234-012f, mds citoMant, Höhr-Grenzhausen, Germany) in a red contra-angle handpiece (high speed) underwater cooling and then with a round bur ISO 10 (Gebr. Brasseler GmbH & Co. KG, Lemgo, Germany) with a green contra-angle handpiece (low speed) until the operator who removed the filling (N.D.) thought having completely removed all composite residues from the cavity. Both the operator (N.D.) and the evaluator (T.G.W.) after restoration removal were blinded to the randomization process. The teeth were weighed again and the volume was determined in the gas pycnometer. Subsequently, all teeth were examined under the microscope with a 25 × magnification (Keyence VHX1000, Neu-Isenburg, Germany) for composite remains and possible dentine fractures and were digitally recorded by the evaluator (Fig. 3). The remaining restoration residues were carefully removed with a round bur (6830L.314.014, Gebr. Brasseler GmbH & Co. KG, Lemgo, Germany) before the second round of the test run (restoration removal) and randomization have been performed. In case of a perforation of the pulp cavity in the first run (*n* = 4), teeth were removed and replaced in the second run. Before the start of the experiment, both the operator and the evaluator underwent theoretical and practical training about the procedure. The evaluator (T.G.W.) has a long experience as a restorative dentist, and he was trained to detect composite remains and dentine fractures under a microscope (25 × magnification, Keyence VHX1000, Neu-Isenburg, Germany). The operator of the present in vitro investigations (N.D.) exhibited routine experience in cavity drilling for treatment purposes and was trained and calibrated by means of 10 sample mandibular teeth before this investigation. The sample teeth were chosen randomly and were discarded after the calibration. The measurements were performed as described previously and repeated one week later again, showing a high correlation for the measurements of weight, volume, and time (intraclass correlation coefficient > 0.8).

### Statistical analysis

Data were inserted in an Excel spreadsheet (Excel™ for Mac 2020, Microsoft Corporation, Redmond, WA, USA). The Wilcoxon signed-rank test was used to evaluate the difference between groups. The difference in weight and volume before and after restoration between groups was assessed with chi-square for proportion. The analysis was performed using Python (Python Software Foundation, DE, USA; https://www.python.org/psf/). The significance level was set after Bonferroni correction at 2.5% (*p* = 0.025).

## Results

The results of weight (g) and volume (cm^3^) before and after removal of the filling using the white-opaque liner and tooth-color liner as well as time (s) for removal of both materials are shown in Table 1.

**Table 1:** Weight and volume before and after restoration removal using the white-opaque liner (case) or tooth-color liner (control) as well as time for removal of both materials

	White-opaque liner	Tooth-color liner
Weight [g]	Mean±SD (*percentage measured after restoration removal*)	
Before	7.57±0.04	7.58±0.07
After	7.54±0.07 (99.60%)	7.52±0.07 (99.21%)
Volume [cm3]		
Before	3.39±0.02	3.40±0.04
After	3.38±0.03 (99.70%)	3.37±0.04 (99.12%)
Time [s]		
Removal	267.10±4.00	264.70±51.00

**Fig. 4:**
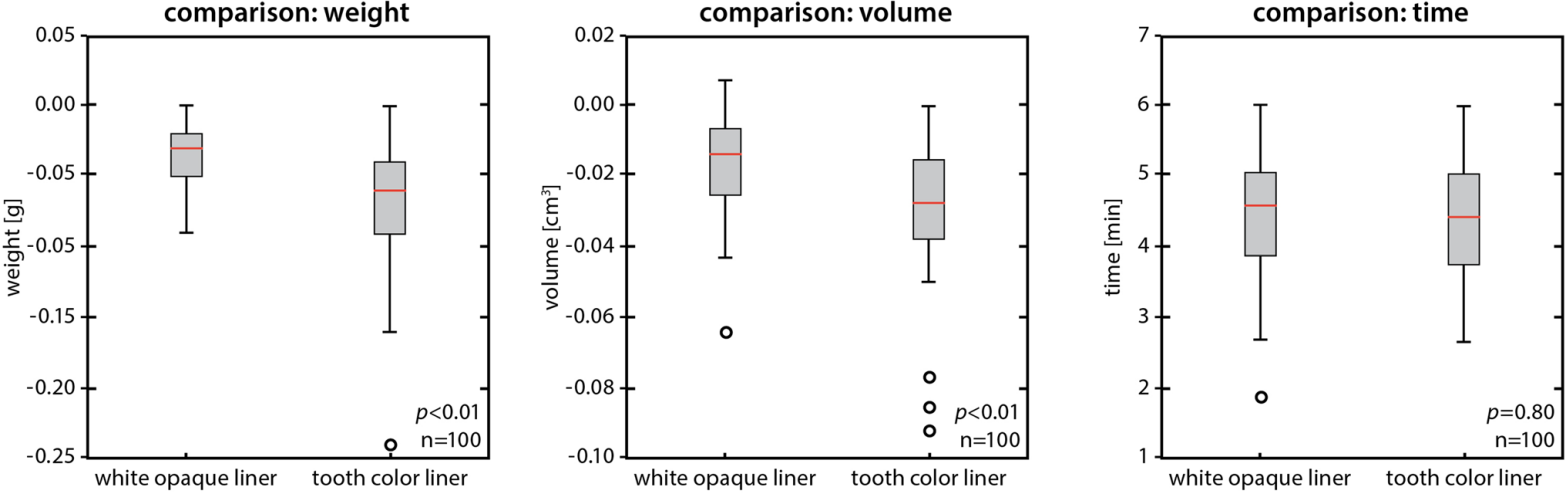
Box plots of case and control groups comparing weight and volume of the tooth substance and time of removal

The difference in the weight of the teeth that were lined with the white-opaque flowable liner was on average − 0.037 g (SD 0.030 g), and the difference in the weight of the teeth that were lined with the tooth-colored flowable composite liner was on average 0.067 g (SD 0.0000 g) (*p* < 0.01) (Fig. 4).

The difference in the volume of the teeth that were lined with the white-opaque liner was 0.016 cm^3^ (SD 0.005 cm^3^). The difference in the volume of the teeth that were lined with the tooth-colored liner was 0.028 cm^3^ (SD 0.003 cm^3^) (Fig. 4) with a statistically significant difference (*p* < 0.01). The measurements of the control group also showed more outliers to significantly lower values. The difference in removal times of the white-opaque liner averaged 267.1 s (4 min and 27.1 s; SD 4 s (0.07 min)). The difference in removal times of the tooth-colored liner was 264.7 s (4 min and 24.7 s; SD 51 s (0.85 min)). No statistically significant difference was detected among the two groups (*p* = 0.80).

During the removal of the restorations, the restoration of the white-opaque liner was totally removed, whereas composite remained from the tooth-colored liner in several cavities (Fig. 3). The pulp chamber was perforated in the bottom of the cavity in a total of six teeth. Four of the perforated teeth belonged to the tooth-colored liner control group and only two to the white-opaque liner group. There was no statistically significant difference between the groups (*p* = 0.80).

## Discussion

This in vitro double-blinded case–control study was aimed to evaluate the efficacy of a white-opaque flowable composite liner used as a depth marking aid to save tooth structure when replacement of a composite restoration in posterior class II teeth is necessary. The white-opaque flowable liner should act as a “stress breaker” and form a bath-shaped floor in the cavity, with margins tapering occlusal and towards the preparation walls to make the cavity lining clearly visible with the white-opaque color of the liner (Fig. 2). Marking the preparation depth with a white-opaque liner allows a significant reduction of tooth structure loss during restoration removal, in terms of both volume and weight of the tooth. On the other hand, no statistical difference was observed regarding the time for the reopening of the cavities between the two groups. Thus, the null hypothesis has been partly rejected for measuring volume and weight for tooth structure loss that was significantly lower using the white-opaque liner (*p* < 0.01). However, the null hypothesis has been accepted for time required to remove the white-opaque liner compared to tooth-colored restoration (*p* < 0.80*)*. Moreover, the pulp chamber floor was perforated only six times, twice as often with tooth-colored restorations than when used with an optical removal aid in the form of a white-opaque liner.

Measurements of tooth structure loss after restoration removals confirm that tooth structure is lost when a restoration is replaced and that the material used also has an influence on this [13, 15, 16]. Composite restorations have the highest volume loss compared to other restorative materials like glass ionomer cement and amalgam [13]. Iatrogenic overextension of the cavities was reported to be up to 37% enlarged [16]. However, when different sizes of class I cavities were prepared in extracted premolars with a depth of 1.5–2.5 mm in the two groups (tooth-colored composite and composite with color variation of three shades), no significant differences were observed [17], but significant tooth structure loss was measured in both groups. The deeper the initial preparation, the greater the loss of tooth structure after restoration removal. In a study on cavity enlargement after restoration removals, the blue-colored build-up composite Rebilda (VOCO, Cuxhaven, Germany) showed better results than a composite matched to the tooth shade with a flowable composite underfilling or photochromic Tetric Ceram Chroma (Ivoclar Vivadent, Schaan, Liechtenstein) [18]. However, a significant minimization of tooth structure loss was achieved by extending the photochromic underfilling not only to the bottom of the preparation but also to the cavity walls. That an optical delineation of the underfilling has a positive effect on the excavated preparation size is confirmed and substantiated by the present study.

Before removal, plasticizers might be added to the composite restoration to chemically weaken the bond between the restoration and the surrounding tooth structure [19] and therefore facilitate the removal of the restoration and protect the preparation from extension. After photographic measurement, the magnification of the cavity was 3–5% [19]. Although the approach of this softening technique seems to be promising, the white-opaque material used in the present study is superior in terms of protection of tooth structure. When comparing the removal of composite and amalgam, the removal of composite from class II cavities resulted in significantly larger preparation volumes compared to the removal of amalgam [15].

The removal of a composite restoration is more time-consuming than that of non-tooth-colored materials, which are visually more distinguishable from tooth substance [13, 16]. The mean removal time for composite restorations is 24 min, significantly longer than the removal of amalgam (15 min) or glass ionomer cement (11 min) [16]. However, data in the literature is very heterogeneous; 7 min for composite removal can also be found [16]. Preparation size plays a significant role. A weakness of the current study is that the procedure was carried out with only a single operator, blinded by randomization, but that operator was able to perform the restorations under optimal light and laboratory conditions. The present outcomes do not allow the exclusion of a systematic bias effect of one operator related to the benefits of the proposed method or attributable to the manual dexterity/skill of the operator. This needs to be further investigated. Furthermore, despite the observed small differences in weight and volume (> 1%), the double use of teeth (two runs of 50 teeth each), which might have only a very small influence that can be neglected, should be mentioned. Nevertheless, practical professional experience as a dentist influences the loss of tooth structure during a restoration removal [20]. Clinical factors such as difficult light and visibility conditions or limited mouth opening, which play an important role in removal time, were also not considered. The obtained results need to be confirmed in a clinical study.

Layer thickness and depth of cure of a composite are related [21]; thick layers cure insufficiently and reduce both mechanical properties and biocompatibility, which is why the increment technique with thinner layers is recommended. Therefore, the white color of the liner used in the present study needs to be discussed. However, as a contrasting color for restorative therapy [13], the white color seems to be the most useful color, because it does not simulate deep caries under restorations and is well-known to all dentists from zinc oxide phosphate cement as an underfilling. Furthermore, the overall esthetic appearance is also least disturbed with white when applied to cavity walls or the proximal box. According to the manufacturer, a high opacity of the colored flowable composite should be realized, since such a material is only applied in a thin layer.

If such a demarcation material had the same opacity as a conventional flowable composite, it would not or only insufficiently be visible on the tooth structure despite its white color. While the setting of a correspondingly high opacity is technically not a problem, the subsequent light curing is. The more opaque a light-curing material is, the smaller the layer thicknesses that can be polymerized in one polymerization cycle. According to the manufacturer, the through-curing of the material is at a layer thickness of up to 1 mm with 40 s light polymerization and in a normal range of curing time.

## Conclusions

Within the limitations of the current randomized double-blinded in vitro study performed by a single operator, it can be concluded without claim of generality that a white-opaque flowable composite:

- May be suitable as an optical aid for depth marking in restorative removal of tooth-colored class II cavities in posterior teeth
- May protect against tooth structure loss, both in terms of weight and volume with an average difference > 1%
- May probably not affect the time needed for restoration removal
